# Identification of Unusual Phospholipid Fatty Acyl Compositions of *Acanthamoeba castellanii*

**DOI:** 10.1371/journal.pone.0101243

**Published:** 2014-07-09

**Authors:** Marta Palusinska-Szysz, Magdalena Kania, Anna Turska-Szewczuk, Witold Danikiewicz, Ryszard Russa, Beate Fuchs

**Affiliations:** 1 Department of Genetics and Microbiology, Maria Curie-Sklodowska University, Lublin, Poland; 2 Mass Spectrometry Group, Institute of Organic Chemistry, Polish Academy of Sciences, Warsaw, Poland; 3 Institute of Medical Physics and Biophysics, Medical Faculty, University of Leipzig, Leipzig, Germany; University of New South Wales, Australia

## Abstract

*Acanthamoeba* are opportunistic protozoan pathogens that may lead to sight-threatening keratitis and fatal granulomatous encephalitis. The successful prognosis requires early diagnosis and differentiation of pathogenic *Acanthamoeba* followed by aggressive treatment regimen. The plasma membrane of *Acanthamoeba* consists of 25% phospholipids (PL). The presence of C20 and, recently reported, 28- and 30-carbon fatty acyl residues is characteristic of amoeba PL. A detailed knowledge about this unusual PL composition could help to differentiate *Acanthamoeba* from other parasites, e.g. bacteria and develop more efficient treatment strategies. Therefore, the detailed PL composition of *Acanthamoeba castellanii* was investigated by ^31^P nuclear magnetic resonance spectroscopy, thin-layer chromatography, gas chromatography, high performance liquid chromatography and liquid chromatography-mass spectrometry. Normal and reversed phase liquid chromatography coupled with mass spectrometric detection was used for detailed characterization of the fatty acyl composition of each detected PL. The most abundant fatty acyl residues in each PL class were octadecanoyl (18∶0), octadecenoyl (18∶1 Δ9) and hexadecanoyl (16∶0). However, some selected PLs contained also very long fatty acyl chains: the presence of 28- and 30-carbon fatty acyl residues was confirmed in phosphatidylethanolamine (PE), phosphatidylserine, phosphatidic acid and cardiolipin. The majority of these fatty acyl residues were also identified in PE that resulted in the following composition: 28∶1/20∶2, 30∶2/18∶1, 28∶0/20∶2, 30∶2/20∶4 and 30∶3/20∶3. The PL of amoebae are significantly different in comparison to other cells: we describe here for the first time unusual, very long chain fatty acids with Δ^5^-unsaturation (30∶3^5,21,24^) and 30∶2^21,24^ localized exclusively in specific phospholipid classes of *A. castellanii* protozoa that could serve as specific biomarkers for the presence of these microorganisms.

## Introduction

*Acanthamoeba castellanii* is a small amoeba that has been isolated from various natural environmental sources such as soil, fresh water, dust, air as well as from anthropogenic ecosystems. This indicates the ubiquitous occurrence of this organism. Its wide distribution in nature brings humans into contact with this protozoan, and massive evidence for this microorganism is provided by the presence of antibodies to *Acanthamoeba* in healthy individuals [1]. *A. castellanii* cause granulomatous amoebic encephalitis (GAE), a fatal infection of the central nervous system (CNS) that occurs in immuno-compromised patients. The amoeba causes also painful keratitis that can result in blindness also occurring in healthy individuals; the infection is often misdiagnosed and difficult to treat (amoebae are resistant to many therapeutic agents) [2], [3], [4]. The limited success in the treatment of GAE is also most likely due to the inability of drugs to cross the blood-brain barrier into the central nervous system (CNS) to target the pathogen. However, phospholipid (PL) analogues such as hexadecylphosphocholine (HPC) possess anti-*Acanthamoeba* properties and have the ability to cross the blood-brain barrier [5], [6], [7].

Many aspects of *A. castellanii* are still unknown. Therefore, we will use here a “non-targeted” approach based on the characteristic lipid composition of this microorganism. In our opinion, lipids serve this approach much better than proteins because lipids enable the comparison of different species (such as mice and humans): the occurrence of the individual (phospho)lipid classes is rather similar between different vertebrates, while that of microorganisms is normally significantly different from mammalians. The plasma membrane of *Acanthamoeba* consists of about 25% phospholipids [6]. A characteristic trait of amoeba phospholipids is the presence of C20 [8], [9], [10] and, recently reported, 28- and 30-carbon fatty acids (FA) [11]. These are quite unusual fatty acyl residues and may be, thus, useful to screen potential host organisms for the presence of this microorganism.

In addition to different mass spectrometric methods, we applied here for the first time high-resolution ^31^P NMR for the quantitative analysis of phospholipids in *A. castellanii* membranes. The advantages of the ^31^P NMR approach include the unequivocal identification of the individual PL species even in relatively complex mixtures (since only a limited number of P-containing resonances need to be assigned) and the lack of solvent signals, which avoids the requirement for their selective suppression. This is a significant advantage regarding quantitative analysis. Theoretically, ^31^P NMR should permit the differentiation between PLs based not only on the headgroup, but also on the linkage type (acyl-acyl-, alkyl-acyl- or alkenyl-acyl) and the acyl chain composition [12]. In practice, however, even if the differentiation between saturated, moderately unsaturated (18∶1 or 18∶2) and highly unsaturated (20∶4 or 22∶6) acyl residues is basically possible, detailed acyl residue analysis fails [13]. NMR is therefore not the method of choice to analyze the fatty acyl distribution within the individual PL classes. However, mass spectrometry, particularly if combined with chromatographic methods such as HPLC or thin-layer chromatography is a powerful analytical tool to provide details of the fatty acyl composition [14], [15], [16]. To combine the advantages of the individual methods, the phospholipid composition of *A. castellanii* cells will be determined by a variety of different analytical techniques, in particular by one-dimensional thin-layer chromatography, HPLC and tandem mass spectrometry with electrospray ionization (ESI) in the positive and the negative ion mode to identify specific fatty acyl residues. Such a detailed characterization of the phospholipid composition of *A. castellanii* cells by combining different analytical tools of lipid characterization is in our opinion very important since using just one method could lead to erroneous or incomplete data.

We suppose that (phospho)lipids possess a significant potential in the rapid diagnosis of *Acanthamoeba* in clinical and environmental specimens because their unusual very long fatty acyl chain pattern is specific of a given microorganism and distinct from vertebrate (phospho)lipids and other microorganisms such as bacteria, viruses and fungi. Since the infection with *A. castellanii* is problematic and often misdiagnosed as bacterial, viral or fungal infection in keratitis, a specific lipid biomarker might help to diagnose and treat an *A. castellanii* infection more efficiently.

Therefore, the aim of this study is to investigate the overall and positional fatty acyl compositions of the phospholipids from *A. castellanii*. In particular, the distribution of the long chain fatty acyl residues was of interest, and it will be explicitly shown that certain fatty acyl residues are exclusively located in one dedicated phospholipid class. Finally, we describe here for the first time unusual, very long chain fatty acids with Δ^5^-unsaturation (30∶3^5,21,24^) and 30∶2^21,24^ localized exclusively in specific phospholipid classes of *A. castellanii* protozoa.

## Materials and Methods

### *A. castellanii* culture

*A. castellanii* (ATCC 3034) was kindly donated by Dr W. Balamuth (Department of Zoology, University of California). The trophozoites of *A. castellanii* were grown as axenic cultures in peptone-yeast extract-glucose (PYG) medium on a shaker incubator set at 120 rpm and 28°C as described previously [11]. The flasks were inoculated with a 3-day-old amoeba culture to obtain an initial population of approximately 5×10^3^ organisms/ml. Amoebae from the early stationary growth phase were harvested by centrifugation at 300×g for 10 min, washed in amoeba saline prepared according to Band [17] and lyophilized.

### Chemicals

All chemicals for mass spectrometry (chloroform and methanol in the highest commercially available purity as well as ammonium acetate), NMR spectroscopy (sodium cholate, EDTA, deuterated water with an isotopic purity of 96.6%), and buffer preparation (NaCl, TRIS) were obtained from Sigma-Aldrich Chemie GmbH (Taufkirchen, Germany). The components for *A. castellanii* cell culture (proteose peptone and yeast extract) were obtained from Becton, Dickinson and Company (Sparks, USA). Chemicals for LC/MS system (hexane, isopropanol, acetonitrile LC grade) and for TLC were obtained from Merck (Darmstadt, Germany).

PLs used as standards were purchased as 10 mg/ml solutions in CHCl_3_ from Avanti Polar Lipids (Alabaster, MA, USA) and used without purification.

### Analysis of phospholipids

Total lipids were extracted using a modified Bligh and Dyer [18] method, i.e. chloroform/methanol/aqueous HCl (1∶2∶0.1, v/v/v). The addition of HCl is important to avoid losses of acidic phospholipids. After separation of the chloroform and the aqueous phases, the chloroform phase was isolated by a glass syringe and evaporated to dryness in a centrifugal evaporator (Jouan, Germany). The precipitate at the interphase between the organic and aqueous layer (primarily proteins) was discarded and not further investigated, so that lipids tightly bound to proteins were excluded from the study. The dried organic phase was purified from lipophilic proteins by an additional extraction with a mixture of hexane∶isopropanol (3∶2, v/v). The extracts were dried under nitrogen before weighing and then dissolved in chloroform (5.6 mg/ml) for further analysis. Lipid classes were separated into neutral and polar fractions on a silica gel column as described previously [19].

Neutral lipids were eluted with 7 ml of chloroform. The phospholipids were eluted with the following solvents: 5 ml of chloroform-acetone (1∶1, v/v), 5 ml of acetone, 3 ml of chloroform-methanol (8∶2, v/v), 4 ml of chloroform-methanol (1∶1, v/v) and 5 ml of chloroform-methanol (1∶50, v/v). The separation was performed twice. Neutral lipid fractions from both separations were discarded. After evaporation of solvents, polar fractions from the second separation were dissolved in chloroform, pooled, and stored at −20°C. The polar lipid fraction was used for ^31^P NMR spectroscopy, thin-layer chromatography (TLC) and mass spectrometry.

To evaluate the reproducibility of the data, all samples were investigated in four independent replicates.

### PL analysis by ^31^P NMR spectroscopy

The dried organic layers were re-dissolved according to [12], [20] in 50 mM TRIS (pH 7.65) containing 200 mM sodium cholate and 5 mM EDTA. After vortexing ^31^P NMR spectra were recorded on 0.5 ml samples in 5 mm NMR tubes on a Bruker DRX-600 spectrometer operating at 242.88 MHz for ^31^P. All measurements were performed using a direct, selective ^31^P NMR probe at 37°C with composite pulse decoupling (Waltz-16) to eliminate ^31^P - ^1^H coupling. Pulse intervals of the order of T_1_ permit the quantitative analysis of PL integral intensities [21].

Other NMR parameters were as follows: acquisition time: 1 s, data size: 8–16 k, 60° pulse (5 µs), pulse delay 2 s and a line-broadening (LB) of 1 Hz. Chemical shifts were referenced to the most intense resonance of diacyl-PC at δ = −0.60 ppm. All peak assignments were confirmed by comparison with the shift of commercially (Avanti Polar Lipids, Alabaster, USA) available PL11. Spectra were processed using the software “1D WIN-NMR” version 6.2 (Bruker Analytische Messtechnik GmbH, Rheinstetten) including the deconvolution (II) routine for peak area determination. Integral intensities of the selected phosphorus-containing metabolites were divided by the total areas of all detected peaks.

### PL analysis by thin-layer chromatography

Phospholipids were separated by one-dimensional TLC on silica gel 60 F254 plates (Merck, 20×20 cm). The plates were washed twice with chloroform/methanol (1∶1, v/v) and activated at 180°C before use. Phospholipids were applied onto the TLC plates and developed with chloroform∶methanol∶acetic acid∶acetone∶water (35∶25∶4∶14∶2) [22]. Lipids were visualized with 5% sulfuric acid in methanol after charring at 140°C for 15 min or with iodine vapor. Phosphorus was detected using the Bochner method [23], carbohydrates were detected with α-naphthol, amino groups with ninhydrin, and quaternary nitrogen (choline) with the Dragendorff reagent [24]. To determine the relative moieties of the individual lipids in the total mixture, the developed TLC chromatograms were visualized with 5% sulfuric acid in methanol, subsequently scanned and the optical densities of the spots integrated using Bio-Profil v. 99.01 (Image Analysis Software). The relative content of each phospholipid class was determined on digitalized densitograms corrected according to calibration curves for authentic standards. Cardiolipin from bovine heart, 1,2-dipalmitoyl-*sn*-glycero-3-phosphoethanolamine, 1,2-dipalmitoyl-*sn*-glycero-3-phosphocholine (Sigma-Aldrich Chemical Co., St. Louis, MO, USA ), 1-hexadecanoyl-2-(9,12-octadecadienoyl)-*sn*-glycero-3-phospho-L-serine and 1-hexadecanoyl-2-(9-octadecenoyl)-*sn*-glycero-3-phosphoinositol, 1-hexadecanoyl-2-lyso-*sn*-glycero-3-phosphocholine, and sphingomyelin 16∶0 (Avanti Polar Lipids, Alabaster, MA, USA) were used as standards.

Phospholipids (about 1 mg) were applied about 1 cm from the bottom of the silica gel plate as a narrow band. The chromatogram was developed with chloroform∶methanol: acetic acid∶acetone∶water (35∶25∶4∶14∶2). Bands were detected by iodine staining and identified by co-migration with the standards. Subsequently, individual lipid bands were scraped off and transferred to screw-capped tubes and extracted from silica gel with a mixture of chloroform/methanol 1∶2 (v/v). The ratio between silica gel and solvent was 1∶9 (m/m).

### Preparation of fatty acid methyl esters and gas chromatographic analysis

Fatty acids were released from the corresponding lipid classes by heating a dried aliquot of each lipid class with 0.8 M NaOH in 50% methanol for 1 h at 80°C. This method provided in our hands better results than applying acidic conditions in the presence of BF_3_. The free fatty acids extracted with CHCl_3_ from the acidified samples were then converted into methyl esters by methanolysis (0.5 M HCl in methanol, 80°C, 1 h). The solution was then cooled to room temperature and the solvent evaporated. Fatty acid methyl esters were recovered by extraction with chloroform/water (1∶2, v/v) and analyzed by gas-liquid chromatography.

GLC-MS was carried out on a Hewlett-Packard gas chromatograph (model HP 5890A) equipped with a capillary column (HP-5MS, 30 m×0.25 mm) and connected to a mass selective detector (MSD model HP5971). Helium (0.7 ml/min) was used as the carrier gas and the temperature program was initially 150°C for 5 min, subsequently increased to 310°C at a ramp rate of 3°C min^−1^, final time of 20 min.

### HPLC MS analysis of phospholipids

Normal phase (NP) and reversed phase (RP) liquid chromatographic methods were tested for identification of phospholipids isolated from *A. castellanii* cells. The NP LC/MS analysis was carried out using a High-Performance Liquid Chromatograph Prominence LC-20 (Shimadzu, Japan) coupled with tandem mass spectrometer 4000 Q TRAP (Applied Biosystems Inc, USA). The mass spectrometer was equipped with an electrospray (ESI) ion source (Turbo Ion Spray) and triple quadrupole/linear ion trap mass analyzer. LC separations were performed using a 4.6×150 mm Zorbax SIL RX (5 µm) column. Solvent A was hexane/isopropanol (3∶2, v/v) and solvent B was isopropanol/hexane/ 5 mM aqueous solution of ammonium acetate (38∶56∶5, v/v/v). The following elution program was used: from 53% B to 80% B in 23 min, 80% B maintained by 4 min, 80% B to 100% B for 9 min and 100% B maintained by 14 min. The flow rate was 1 ml/min.

RP LC/MS analysis as a complementary method for the identification of PI and PC species was done using a Finnigan LCQ Advantage Max ion-trap mass spectrometer (ThermoElectron Corporation, San Jose, CA, USA) with ESI source. The phospholipid separation was carried out on a cyano column (5 µm, 4.6 mm×250 mm). Milli-Q water (component A), acetonitrile (component B), 0.1% w/v formic acid in acetonitrile (component C) and 1% w/w ammonia in acetonitrile (component D) were used as the mobile phase. The following gradient was employed for LC lipid separation: 12 min isocratic elution using a mixture composed of 40% A, 50.5% B, 9.2% C, 0.3% D and then a linear gradient of B up to 89.5% at the expense of A over 8 min. The final mobile phase composition (0.5% A, 89.5% B, 9.2% C, 0.3% D) was held for 40 min. The eluent flow rate and the column temperature were 0.5 ml min^−1^ and 55°C, respectively.

The low resolution spectra and collision induced decomposition (CID) spectra of identified compounds (the corresponding parent ions were isolated by using optimized settings of the ion trap) were performed in the positive and the negative ion mode. Nitrogen was used as the nebulizer, curtain and collision gas. The tip voltage was kept at 5500 V and -4500 V in the positive and the negative ion mode, respectively. The declustering potential was set to 30 V in both modes. The CID spectra were recorded at collision energies ranging between 35–60 eV.

## Results

The chloroform-methanol extractable lipids of *A. castellanii* grown on the PYG medium accounted for 14.5% of the cell material (122 mg dry weight), out of which polar lipids (56 mg) constituted 46% of the total lipids. The polar lipids were used for ^31^P NMR spectroscopy, thin-layer chromatography (TLC) and LC/MS analyses.

**Figure 1:**
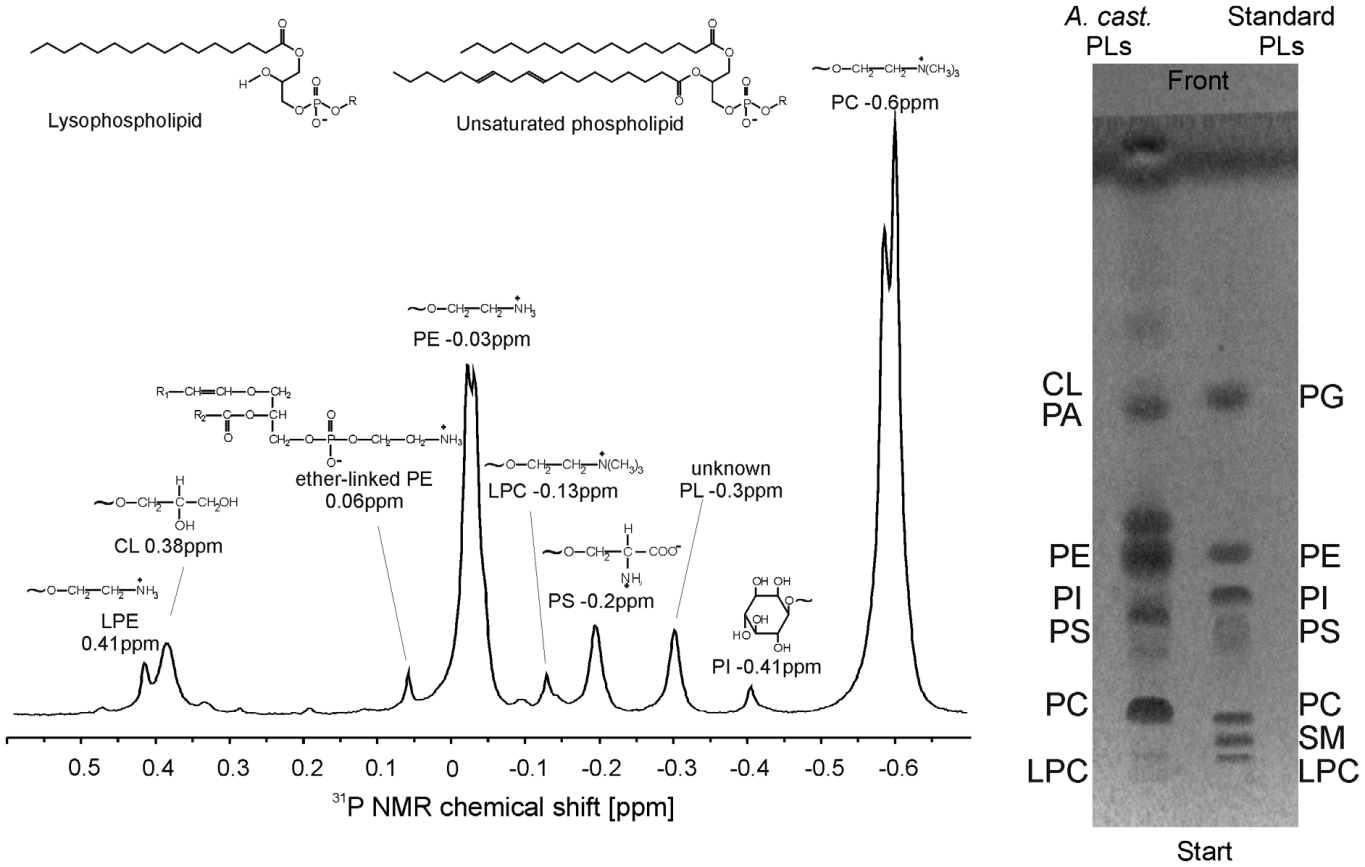
31P NMR spectrum and one-dimensional TLC profile of *A. castellanii* phospholipids. Left: 242.88 MHz ^31^P NMR spectrum of the organic extract of *A. castellanii*. Assignments and chemical shifts are indicated in the spectrum. The chemical structures of a phospholipid and a lysophospholipid is shown. R stands for the different head groups, while R_1_ and R_2_ denote the different carbon chains in the *sn*-1 and *sn*-2 position of the glycerol back bone. Chemical structures of the head groups are shown on top of the corresponding peak. Right: One-dimensional TLC profile of *A. castellanii* phospholipids in comparison with a standard PL mixture that contains the same amounts of all indicated PL. TLC plates were developed in a solvent mixture consisting of chloroform∶methanol∶acetic acid∶acetone∶water (35∶25∶4∶14∶2, v/v/v/v/v). Abbreviations used in peak/spot assignments: CL, cardiolipin; LPC, lyso-phosphatidylcholine; LPE, lyso-phosphatidylethanolamine; PA, phosphatidic acid; PC, phosphatidylcholine; PE, phosphatidylethanolamine; PG, phosphatidylglycerol; PI, phosphatidylinositol; PS, phosphatidylserine; SM, sphingomyelin. For details see text.

The organic extracts of *A. castellanii* were analyzed by ^31^P NMR spectroscopy in order to be able to quantify all PL classes. A selected ^31^P NMR spectrum of *A. castellanii* is shown in **Fig. 1** (left hand). Peak assignments and the corresponding chemical shifts are given on the top of the spectra. This spectrum exhibits signals of nine different phospholipid classes comprising phosphatidylcholine (PC, δ = −0.60 ppm), phosphatidylinositol (PI, δ = −0.41 ppm), phosphatidylserine (PS, δ = −0.2 ppm), lysophosphatidylcholine (LPC, δ = −0.15 ppm), phosphatidylethanolamine (PE, δ = −0.03 ppm), ether-linked glycerophosphoethanolamine (ether-GPE, δ = 0.06 ppm), cardiolipin (CL, δ = 0.38 ppm), and lysophosphatidylethanolamine (LPE, δ = 0.41 ppm). Although these resonances could be unequivocally assigned, another resonance was detected at δ = −0.3 ppm that could not be convincingly characterized. However, it is claimed that this resonance is caused by a PI species with a very remarkable fatty acyl composition. However, a more detailed structure elucidation would go beyond the scope of this manuscript.

Quantitative ^31^P NMR data were obtained by integration of the individual ^31^P NMR resonances: PC (47.5±2%), PE (29±2%), PS (5.6±0.2%), and CL (6.9±0.3%) were the most abundant phospholipids. Other minor phospholipids comprised PI (1.2±0.05%), LPC (1.8±0.1%), LPE (1.5±0.05%), and ether-GPE (1.7±0.08%).

### TLC analysis

**Fig. 1** shows the one-dimensional TLC profile of the phospholipids of *A. castellanii* (right hand side), displaying seven bands. Individual polar lipid components were identified by comparison of their R_f_ values with those of the authentic standards and specific staining properties.

The following components were identified in the seven bands:

Band 1 contained lysophosphatidylcholine (LPC) and band 2 phosphatidylcholine (PC) as revealed by staining with the Dragendorff reagent and co-migration with standards. The predominant fatty acids obtained by hydrolysis of the PC fraction were octadecanoic (33%) and octadecenoic (25%) acid, as shown by GC/MS analysis of band 2 extracted from silica gel. However, long chain unsaturated FAs could also be detected in the PC fraction: eicosatetraenoic (20∶4 Δ^5,8,11,14^), eicosatrienoic (20∶3 Δ^8,11,14^) and eicosadienoic (20∶2 Δ^11,14^), which constituted 19 mol% of the PC species (**Table 1**). The fatty acids of LPC were primarily hexadecanoic (41 mol%) and octadecanoic (29 mol%) acid.

**Table 1:** Content (given in mol %) of the fatty acyl residues of each phospholipid class of *A. castellanii*.

Fatty acid	LPC	PC	PS	PI	PE	PA	Cardiolipin
14∶0	13	4	7	2	6	26	6
16∶1 Δ^7^ *	8	4	5	11	5	6	4
16∶0	41	9	19	36	13	28	17
17∶0	tr	1	1	1	1	1	1
18∶2 Δ^9,12^	tr	5	9	2	5	5	5
18∶1 Δ^9^	9	25	31	19	21	17	28
18∶0	29	33	12	28	29	15	28
20∶4 Δ^5,8,11,14^		7	1	tr	4		tr
20∶5		tr	tr		tr		tr
20∶3 Δ^8,11,14^		7	4	tr	4	tr	2
20∶2 Δ^11,14^		5	6	1.0	6	2	3
20∶1			tr		tr		
28∶2 Δ^5,21^							tr
28∶1 Δ^21^			1		2	tr	2
28∶0			1		1		2
30∶3 Δ^5,21,24^			1		1		2
30∶2 Δ^21,24^			2		2	tr	tr
sum of saturated	83	47	40	67	50	70	54
sum of unsaturated	17	53	60	33	50	30	46

Data were determined by GC/MS analysis of the FAs methyl esters subsequent to alkaline hydrolysis of the corresponding lipid class. Standard deviations of all measurements are estimated to be of the order of ±5%.

*The position of double bonds was already determined in a previous paper [11], tr – trace (<0.5%).

Band 3 contained phosphatidylserine (PS). This phospholipid stained positively with ninhydrin and phosphomolybdate. This phospholipid exhibited a higher content of unsaturated (60 mol%) than saturated fatty acids. Hexadecanoic and octadecanoic acid, which were less abundant in the other PL classes, were the dominant saturated acids, while octadecenoic acid (31 mol%) was the main unsaturated one in the PS fraction. Additionally, there were also C20 acids: eicosatetraenoic (20∶4 Δ^5,8,11,14^), eicosatrienoic (20∶3 Δ^8,11,14^) and eicosadienoic (20∶2 Δ^11,14^), C28 acids - octacosenoic (28∶1 Δ^21^) and C30 acids - triacontadienoic (30∶2 Δ^21,24^) and triacontatrienoic (30∶3 Δ^5,21,24^) acids (**Table 1**).

Band 4 contained phosphatidylinositol (PI). This phospholipid was identified by staining with phosphomolybdate and co-migration with the standard. Saturated acids, mainly hexadecanoic and octadecanoic acid, dominated in this phospholipid class.

Band 5 corresponding to phosphatidylethanolamine (PE) was identified by its characteristic R_F_ value and staining with ninhydrin and phosphomolybdate. Octadecanoic acid was predominant in this class. Unsaturated acids constituted approximately half of the total FAs (50 mol%). As in the case of PS, PE contained 20-, 28-, and 30-carbon acids, with only 2 mol% of octacosenoic (28∶1 Δ^21^) and 2 mol% of triacontadienoic (30∶2 Δ^21,24^) acid.

Band 6 contained phosphatidic acid (PA). PA was identified by its position relative to other lipids, the co-elution with the standard, and the absence of ninhydrin staining. PA belongs to the least abundant phospholipids and was presumably therefore not detectable by ^31^P NMR. PA was characterized by the presence of saturated tetradecanic (26 mol%) and hexadecanoic (28 mol%) acid. The GC/MS analysis of the methyl esters demonstrated also minor amounts of octacosenoic (28∶1 Δ^21^) and triacontadienoic acid (30∶2 Δ^21,24^), which were also identified by LC/MS.

Band 7 represented cardiolipin (CL), which was phosphomolybdate-positive. Cardiolipin included considerable amounts of octadecanoic (18∶0) and octadecenoic (18∶1 Δ^9^) acids. Moreover, CL contained eicosatrienoic (20∶3 Δ^8,11,14^) and eicosadienoic (20∶2 Δ^11,14^), as well as octacosenoic (28∶1 Δ^21^), octacosanoic (28∶0) and triacontatrienoic (30∶3 Δ^5,21,24^) residues.

In order to confirm and extend the TLC analysis of the polar lipids of *A. castellanii*, all identified phospholipid classes were additionally analyzed by LC/MS.

### HPLC/MS analysis

Normal (NP) and reversed phase (RP) liquid chromatography coupled with mass spectrometry detection were used to perform a more detailed analysis of the individual PL species of *A. castellanii* cells. The NP LC/MS method facilitates separation of the PLs into their respective classes and enables the characterization of the individual molecular species (data not shown). This is an indispensable method for PL identification, particularly for complex extracts from biological sources, where many different lipid classes with strongly varying fatty acyl compositions can be expected. In contrast to NP LC/MS, RP LC/MS analysis is capable of separating PLs according to their fatty acyl compositions. Although it is a useful tool for the identification of the individual molecular species in isolated phospholipid fractions, in this study the total mixture of PA, PS, PE, PC and PI was directly analyzed by RP LC/MS technique.

### Identification of phospholipid classes

Phospholipid classes were identified by the NP LC/MS method using mainly precursor ion scan (PI) and neutral loss scan (NL) techniques as recently essentially reviewed [25]. These scanning methods can be utilized to analyze selectively dedicated phospholipid headgroups in a mixture. Defined phospholipid standards were also used to determine the retention time and fragmentation patterns of the PLs of interest.

In the precursor ion scan, the ion at *m/z* 184 (positive ion mode) corresponding to protonated phosphocholine was used to identify the individual PC species in the sample (data not shown). In contrast, phosphatidic acids were analyzed in the negative ion mode by the characteristic fragment at *m/z* 153 corresponding to deprotonated phosphoglycerol.

In the neutral loss scan technique, the tendency of PS and PE species to loose serine (87 Da) or phosphoethanolamine (141 Da), respectively, was used to identify these PL classes in the positive ion mode.

In contrast to other PL classes, PI species were identified by the RP HPLC/MS method only. Using NP HPLC conditions (described in Materials and Methods) exclusively the monophosphorylated but not diphosphorylated derivatives of inositol-containing lipids were identified. The detailed structural characterization of these compounds was beyond the scope of this study.

### Identification of individual phospholipids

Many PL classes possess a net negative charge at neutral pH. PC and PE molecules are zwitterionic ions; therefore, positive and negative-ion mass spectra of these PLs are accessible. PI and PS species also can be analyzed in the positive ion mode giving the sodiated and the protonated adducts, whereas in the negative ion mode deprotonated PI and PS are directly observed [26].

In this work, low-resolution MS spectra of individual PL classes were recorded in the positive and the negative ion mode. In the negative ion mode, deprotonated PE, PS, PI and PA species were directly observed while for PC-containing compounds the ions [M+CH_3_COO]^−^ were also detectable. In the positive ion mode, the mass spectra of PC, PE and PS were dominated by the protonated species.

To determine the structures of the analyzed compounds, CID spectra of individual PLs were performed and compared to the CID spectra of the PL standards and literature data [26]. Since the fragment ion spectra recorded in the negative ion mode were easier to interpret (due to the reduced interference of the different adducts obtained in the positive ion mode), these CID spectra mainly served to establish the structures of the analyzed PLs (**Table 2**).

**Table 2:** Survey of the most abundant molecular species of *A. castellanii* phospholipids determined by LC/MS operating in the negative (−) and positive (+) ion mode.

PL class	m/z	Fatty acyl composition
**LPC**	480.2 (+)	16∶0
	506.2 (+)	18∶1
	508.3 (+)	18∶0
**PC**	750.8 (−)^a^	16∶0/14∶0
	758.6 (+)	16∶1/18∶1^c^
	760.6 (+)	16∶0/18∶1^c^
	764.8 (−)	17∶0/14∶0^c^
	778.8 (−)	14∶0/18∶0
	784.6 (+)	18∶1/18∶2
	786.6 (+)	18∶1/18∶1
	788.6 (+)	18∶0/18∶1
	806.6 (+)	18∶1/20∶5^c^
	812.6 (+)	18∶1/20∶2
	816.4 (−)	14∶0/20∶2; 16∶1/18∶1
	846.4 (−)	18∶0/18∶1
	850.8 (−)	18∶2/20∶4
	852.8 (−)	18∶1/20∶4
	854.8 (−)	18∶0/20∶4
	854.7 (−)	18∶1/20∶3
	856.8 (−)	18∶0/20∶3
	858.9 (−)	18∶0/20∶2
	866.5 (−)	18∶2/20∶3; 18∶1/20∶4
	868.7 (−)	18∶0/20∶4^c^
**PE**	688.4 (−)	14∶0/18∶1
	714.5 (−)	16∶1/18∶1; 16∶0/18∶2; 14∶0/20∶2
	716.4 (−)	16∶0/18∶1
	742.5 (−)	16∶0/20∶2; 18∶0/18∶2; 18∶1/18∶1
	744.5 (−)	18∶1/18∶0
	764.8 (−)	18∶1/20∶4; 18∶2/20∶3
	766.8 (−)	18∶2/20∶2; 18∶1/20∶3; 18∶0/20∶4
	768.8 (−)	18∶0/20∶3; 18∶1/20∶2
	768.7 (+)	18∶0/20∶4
	**882.6 (−)**	**30∶2/16∶0**; **28∶1/18∶1** b
	**908.6 (−)**	**28∶1/20∶2**; **30∶2/18∶1**
	**910.6 (−)**	**30∶1/18∶1**; **28∶0/20∶2**
	**930.6 (−)**	**30∶2/20∶4**
	**932.9 (+)**	**30∶3/20∶3**; **20∶4/30∶2**
	**934.6 (−)**	**30∶2/20∶2**
	938.5 (+)	**20∶1/30∶2**
**PS**	758.7 (+)	16∶1/18∶1^c^
	804.6 (−)	18∶2/20∶5
	806.4 (−)	18∶2/20∶4; 18∶1/20∶5
	808.5 (−)	18∶1/20∶4
	810.7 (−)	18∶1/20∶3^c^
	812.9 (−)	18∶0/20∶3
	812.6 (+)	18∶1/20∶2^c^
	**972.6 (−)**	**30∶3/20∶4**
	**978.6 (−)**	**30∶2/20∶2**
**PI**	831.5 (−)	16∶1/18∶2
	833.5 (−)	16∶1/18∶1
	835.5 (−)	16∶0/18∶1
	847.4 (−)	17∶0/18∶2
	859.5 (−)	18∶1/18∶2
	863.6 (−)	18∶1/18∶0
	889.6 (−)	18∶0/20∶2
**PA**	645.2 (−)	16∶0/16∶1
	661.5 (−)	15∶0/18∶1
	673.5 (−)	16∶0/18∶1
	675.5 (−)	16∶0/18∶0
	689.9 (−)	18∶0/17∶0^c^
	695.5 (−)	18∶1/18∶2
	**865.6 (−)**	**18∶1/30∶2** c
	**865.6 (−)**	**28∶1/20∶2** c

a Phosphatidylcholines were detected as acetate [M+CH_3_COO]^−^ or formate [M+HCOO]^−^ adducts.

b bold-print - PL with long chain FAs.

c PL species without assignments to *sn-1/sn-2* position.

Loss of fatty acids as neutral molecules or as ketenes occurs readily during the fragmentation of PLs. The high intensities of ions corresponding to lysophospholipid-like products ([M-H-RCOOH]^−^ or [M-H-R′CH = C = O]^−^ and anions of free fatty acids ([RCOO^−^]) have been suggested to be useful for the determination of the *sn*-1 and *sn*-2 positions of the fatty acyl residues in the individual PL [26]. In this work, the following rule was adopted for positional identification of the acyl residues: for PC-, PE- and PI-derived lipids, the ions corresponding to neutral losses of the *sn*-2 substituents (as a FA and a ketene) are more abundant than the ions reflecting neutral losses of the analogous substituents from the *sn*-1 position. In the case of PS- and PA-derived PL, the carboxylate anion arising from the *sn*-1 FA is more prominent than the carboxylate anion derived from the *sn*-2 FA.

To identify the positions of the fatty acyl residues in the PL structures, the CID spectra of the analyzed PLs with well-defined fragment ions were chosen (**Table 2**).

**Figure 2:**
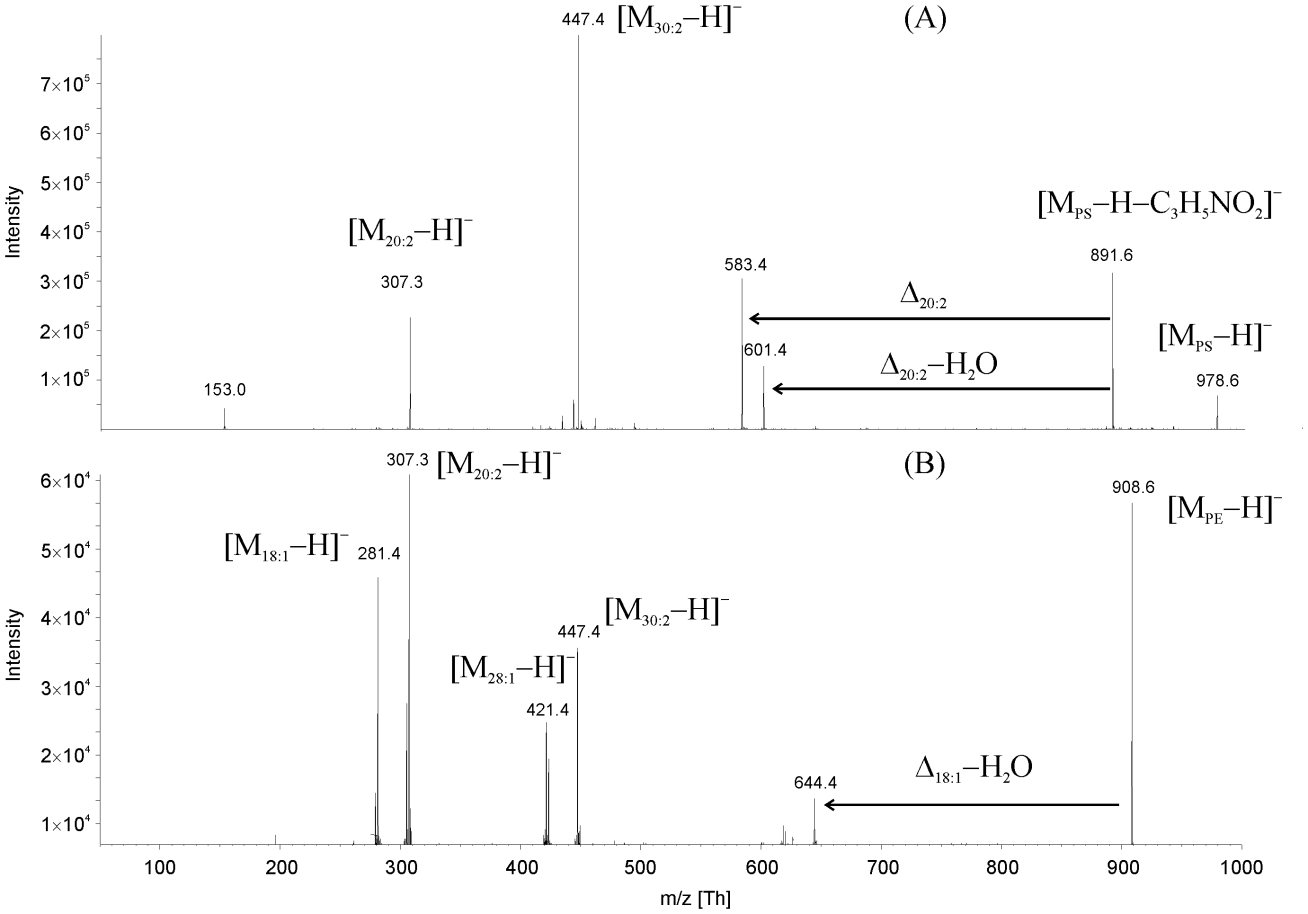
CID spectrum (recorded in the negative ion mode) of (A) a dedicated PS containing 30∶2 (*sn*-1) and 20∶2 (*sn*-2) fatty acyl residues. Δ 20∶2 elimination of eicosadienoic acid, Δ 20∶2-H_2_O–elimination of eicosadienoic ketene and (B) a given PE species with the following fatty acyl combinations: 28∶1/20∶2; 30∶2/18∶1.

Most PC species were identified by HPLC/ESI-MS. This is consistent with the TLC results, which showed that PC is the main polar lipid in the PL extract of *A. castellanii* cells. PC species containing mainly octadecanoyl or octadecenoyl residues were detected. **Fig. 2** shows the negative ion fragmentation spectra of PS (trace A) and PE (trace B) species containing 30∶2 (*m/z* 447.4), 28∶1 (*m/z* 421.4) and 20∶2 (*m/z* 307.3) fatty acids as representative examples. The loss of eicosadienoic and octadecenoic acids as neutral molecules or ketene is obvious from these CID spectra. These data help to unequivocally identify a given lipid.

Regarding the PA and PI classes, molecular species with pentadecanoyl, hexadecanoyl and heptadecanoyl residues were identified. The 30- and 28-carbon fatty acids were found particularly in combination with octadecenoic and eicosadienoic acids, respectively in PA-containing compounds. The PE class was dominated by octadecenoic and octadecadienoic acids. Thus, there are significant differences between the different PL classes in the content of long and short chain fatty acyl residues.

## Discussion

Upon separation into neutral and polar fractions using the Bligh and Dyer extraction method and analysis of the polar fractions by ^31^P NMR spectroscopy, 1D TLC and LC/MS, the following PL classes could be identified in *A. castellanii*: PC, PE, PS, PI, LPC, cardiolipin (not discussed in this manuscript) and PA. The PL composition was thus similar to that of the Neff *A. castellanii* strain obtained by Ulsamer *et al.* [8]. However, slight quantitative differences were observed. The two predominant lipid classes were PC and PE accounting for approximately 75% of the total PL. A significant content of these PL is characteristic of most parasitic protozoans [27].

Seventeen different C14-C30 FAs of *A. castellanii* were identified by GC-MS analyses of their methyl esters. They included both saturated FAs such as C14∶0, C16∶0, and C18∶0 as well as unsaturated FAs: C18∶1 Δ^9^, 18∶2 Δ^9,12^, C20∶4 Δ^5,8,11,14^, C20∶3 Δ^8,11,14^, and C20∶2 Δ^11,14^. The investigated PLs also contained long-chain C28 and C30 acyl residues. These long chain fatty acids are a characteristic feature of *A. castellanii* since PL of most multicellular organisms contain nearly exclusively C16-22 FAs. However, very long-chain C23-32 FAs were also found in marine sponges [28]. The 30∶2 Δ^5,9^ acid is present in *Cinachyrella schulzei*, and 30∶3 ^Δ5,9,23^ in *Chondrilla nucula*. Although the chain length and the number of double bonds are comparable, the corresponding *A. castellanii* fatty acids differ regarding their positions of the double bonds.

Fatty acid analysis of the individual phospholipid fractions provided evidence that certain long chain FAs of *A. castellanii* are associated with specific phospholipids. The presence of the 28- and 30-carbon acids was confirmed in PE, PS, PA and cardiolipin. PLs with the compositions 28∶1/20∶2, 30∶2/18∶1, 28∶0/20∶2, 30∶2/20∶4 and 30∶3/20∶3 were mainly identified in the PE fraction. They were less abundant in PS species such as 30∶2/20∶2, 30∶3/20∶4. The specific location of long chain fatty acids in certain PLs has been observed also in sponges [28], where the PE fraction contains large amounts of 26∶2 and 26∶3, for instance, in *M. prolifera* and *Halicondria panacea*. Most of the 30∶3 fatty acids in *C. nucula* are also present in PE. PS contains high levels of 26∶2 and 26∶3 in *M. prolifera* and large amounts of 30∶4 and 30∶5 in *C. celata* while only small amounts of 30∶3 Δ^5,9,23^ were detectable in *C. nucula* [29].

The LC/MS/MS analysis of the fatty acyl distributions in *A. castellanii* PLs confirmed the general principle that the *sn*-1 position is usually occupied by a saturated or monoenoic residue, whereas polyunsaturated residues are normally located in the *sn*-2 position of mammalian PLs. However, in the case of selected *A. castellanii* PLs, the *sn*-1 position contains a higher unsaturated fatty acyl residue, for example, PE 18∶2/20∶2, 30∶2/20∶4, 30∶3/20∶3 and PS 30∶2/20∶2, 18∶2/20∶4, 30∶3/20∶4. It seems likely this high double bond content affects the replication of *A. castellanii* in the same way as it affects the development of other organisms. For instance, spermatozoa are also characterized by a considerable content of unsaturated fatty acyl residues within their phospholipids because this is a prerequisite for successful fusion with the female oocyte [30]. Furthermore, the high level of unsaturation of *A. castellanii* membranes affects their fluidity significantly and, thus, is a prerequisite of effective phagocytosis. Acanthamoeba phagocytosis may be both, an efficient way of obtaining nutrients for amoeba, and a significant aspect regarding the pathogenesis of acanthamoeba infections [31].

In a nutshell our findings have proven that the PL class composition of *A. castellanii* does not differ significantly from the PL class composition of mammalian cells [32] or other parasitic protozoan. However, we have also shown that *A. castellanii* has not only an unusual fatty acyl composition but also an unusual distribution of these fatty acyl residues in the glycerol backbone of selected phospholipids. We describe here for the first time unusual, very long chain fatty acids with Δ^5^-unsaturation (30∶3^5,21,24^) and 30∶2^21,24^ localized exclusively in specific phospholipid classes of *A. castellanii* protozoa. This unusual fatty acyl composition and distribution could serve as a biomarker for the identification of *A. castellanii*. Since the majority of potential hosts are not expected to possess such long fatty acyl residues, the presence of *A. castellanii* can be easily screened by the presence of these characteristic fatty acids. Little is so far known about the distribution of *A. castellanii* within the body of the affected host and further studies are needed to clarify this aspect. As far as we can say, the following biopsy materials from infected patients might useful to check for the presence of typical *A. castellanii* fatty acids: corneal scrapings, the content of the nasal cavity, the throat, the internal ear, skin, as well as cerebrospinal fluid. It can be expected that *A. castellanii* lipids will be present only to a very small amount in physiologically relevant samples. Therefore, it is presumably advantageous to use the high sensitivity and the significant resolving power of GC/MS to screen selected body fluids and/or tissues (vide infra) for their contents of unusual very long chain, polyunsaturated free fatty acids (such as 30∶3 and 30∶2) which are characteristic of *A. castellanii* cells. A detailed lipidomics analysis of the intact (phospho)lipids would, however, provide much more information because the intact lipids can be analyzed. In contrast, all lipids have to be hydrolyzed and derivatized if GC/MS analysis is performed. Therefore, all information regarding the fatty acyl compositions of the different (phospho)lipid classes is lost.

As the unusual very long fatty acyl chain composition of A. castellanii are presumably absent in vertebrate, bacterial, viral and fungal phospholipids, this may have significant potential regarding the rapid diagnosis of *Acanthamoeba* in clinical and environmental specimens.

Finally, since synthetic PC analogues were recently suggested as promising drugs against *Acanthamoeba* species [7], [33], the results obtained may help to find more effective antimicrobials to treat *Acanthamoeba* infections. The goal of our future studies is to assess the biological significance of these fatty acids and their sensitivity/resistance to antiprotozoal agents.
